# The Transformative Effect of Training on Informed Consent: A Comparative Study Among Medical Postgraduate Researchers

**DOI:** 10.7759/cureus.97987

**Published:** 2025-11-27

**Authors:** Mehdi A Mirza, Swathi Suravaram, Imran A Siddiqui

**Affiliations:** 1 Pharmacology, Employees' State Insurance Corporation (ESIC) Medical College and Hospital, Hyderabad, IND; 2 Microbiology, Employees' State Insurance Corporation (ESIC) Medical College and Hospital, Hyderabad, IND; 3 Biochemistry, Employees' State Insurance Corporation (ESIC) Medical College and Hospital, Hyderabad, IND

**Keywords:** icmr guidelines, informed consent, postgraduate medical education, research ethics, training

## Abstract

Background: Inadequate implementation of the informed consent (IC) process remains a major ethical and regulatory challenge, particularly among postgraduate (PG) students conducting thesis research. This study aimed to assess the impact of a structured training program on the IC documentation and practices of medical postgraduates.

Methods: This prospective cohort, comparative study included 60 PG students from two consecutive batches: an untrained batch (n=30) and a trained batch (n=30). The trained batch underwent a 9-hour, three-session formal training on IC practices aligned with the Indian Council of Medical Research (ICMR) 2017, New Drugs and Clinical Trials (NDCT) 2019, and ICH-Good Clinical Practice (GCP) guidelines. Informed consent forms (ICFs) and the consent process itself were evaluated using a 30-item checklist across five domains: Content Quality, Counseling Process, Comprehension Assessment, Documentation Standards, and Regulatory Compliance. Data was collected via document review and direct observation by independent investigators. Comparative analysis used the Chi-square and independent t-test (p < 0.05).

Results: The mean overall checklist score was significantly higher in the trained batch (26 ± 2, 86%, Excellent grade) compared to the untrained batch (15 ± 3, 51%, Satisfactory grade) (p<0.05). Domain-specific analysis showed dramatic improvement in the trained group, notably in Comprehension Assessment (92% scoring ≥80% vs. 13% in the untrained group) and ICF/Informed Consent Document (ICD) Content Quality (95% scoring ≥80% vs. 8%). Deficiencies in the untrained group included absent comprehension checks, lack of compensation clauses, and poor regulatory compliance (e.g., 0% for re-consenting after protocol amendments).

Conclusion: A structured, comprehensive training significantly and immediately improves the quality of IC documentation and practices among medical postgraduates, moving performance from a “Satisfactory” to an “Excellent” standard. Integrating such targeted, hands-on training into the PG curriculum is essential for enhancing ethical standards in clinical research.

## Introduction

Informed consent (IC) stands as a basis of ethical research, safeguarding the autonomy, rights, and well-being of research participants [1]. For Doctor of Medicine (MD) and Master of Surgery (MS) postgraduate (PG) students conducting thesis-based research involving human subjects, obtaining IC is not just a regulatory requirement, but it is a reflection of ethical integrity and professionalism in clinical research [2]. Despite its importance, numerous global, regional, and local studies have shown that the informed consent process is often inadequately implemented, with common shortcomings in documentation, content clarity, counseling practices, and participant comprehension [2-7].

In India, the ethical framework governing research involving human participants is well-established, with detailed guidance provided by the Indian Council of Medical Research (ICMR) 2017 guidelines, the New Drugs and Clinical Trials (NDCT) Rules 2019, and the Indian Good Clinical Practice (GCP) Guidelines [5,8-10]. These regulations emphasize essential components of the informed consent process, including the preparation of a clear and comprehensive Informed Consent Form (ICF) and Informed Consent Document (ICD), counseling of participants in a language they understand, assessment of comprehension and proper documentation. However, as per the internal ethics quality improvement program, it was brought to focus that the compliance with these guidelines among PG students conducting research was often inconsistent. There is a need for enhanced training and education in medical teaching institutes to improve ethics in research [11].

Several factors contribute to this inconsistency, such as a lack of awareness about the ethical and regulatory frameworks, inadequate training in research ethics, poor mentorship, and a mechanical or checkbox approach to consent [12-15]. This raises serious concerns about the ethical conduct of research at the postgraduate level, especially when participants are recruited from vulnerable populations in clinical settings [16]. Questionable research practices were observed among student researchers due to different levels of knowledge and varying attitudes toward research ethics [11]. There is a need in institutions to deliberately cultivate and refine the understanding of research ethics among PG students to enable compliance with national regulatory rules and guidelines.

Recognizing this gap, medical PG training institutions have implemented structured training sessions to educate PG students on the ethical, legal, and procedural aspects of IC in research [17]. These sessions are designed to foster a deeper understanding of the components of informed consent, including the design and content of the ICF/ICD, patient counseling, comprehension verification, voluntary participation, documentation of the process, and compliance with national and international standards [18]. While such training programs are increasingly recommended, their real-world impact on the IC practices of medical postgraduates remains underexplored.

This study was conceptualized to fill that gap by assessing the influence of training on the IC practices of medical postgraduates involved in thesis-based research. It compared the practices of postgraduate batches that did not receive any structured training with a batch that underwent comprehensive training sessions covering all aspects of the informed consent process aligned with GCP, ICMR 2017, and NDCT Rules 2019. Through a comparative design, this study evaluates critical aspects of the informed consent process, such as the preparation and completeness of ICF and ICD, adequacy of counseling, assessment of participant understanding, documentation standards, and procedural consistency. By highlighting the variations in practices between trained and untrained groups, the study aims to determine whether training can bridge the gap between theoretical ethical standards and practical implementation.

## Materials and methods

Study design and setting

This was a prospective cohort comparative study conducted in a 1000-bedded Government Medical College and Postgraduate Institute with broad and super-specialty tertiary care disciplines. The institution is recognized by the National Medical Commission (NMC) of India. The study aimed to evaluate the impact of structured training on IC documentation and practices among postgraduate (PG) medical students involved in thesis-based research. The study compared the objective outcomes of an untrained batch (2023-2024) with a trained batch (2024-2025) using a 30-item checklist based on national (ICMR, NDCT) and international (ICH-GCP) guidelines. The trained batch received formal training on research ethics and the IC process before initiating their thesis work.

Study Population and Sampling

The study population included PG students from two consecutive academic years, i.e., 2023-2024 and 2024-2025. All students who submitted their thesis proposals for conducting prospective observation or intervention research to the Institutional Ethics Committee from the selected batches were eligible. The students who had dropped out, changed thesis topic, conducted retrospective medical record-based research, applied for IC waiver, and whose thesis did not involve human participants were excluded.

Sixty-three (11 intervention and 52 observation) out of 83 students from the 2023-2024 and 68 (9 intervention and 59 observation) out of 87 postgraduates from the 2024-2025 batch were considered eligible.

A simple random sampling method was used. Individually, each of the 63 students from the 2023-2024 batch and 67 students from the 2024-2025 batch were assigned a unique identity number. Thirty students were randomly selected for each batch using the Excel RANDBETWEEN function. The total sample size was 60, comprising 30 students in the untrained batch and 30 students in the trained batch.

Data collection tools and parameters

A structured data extraction form and a self-developed checklist (Provided as a supplementary file), formulated with reference to the ICMR National Guidelines for Biomedical and Health Research (2017), the New Drugs and Clinical Trials Rules (2019), and the ICH-GCP guidelines, were employed to evaluate informed consent practices among postgraduate students. The specific domains and components assessed are summarized in Table 1.

**Table 1:** Domains for Evaluating the Informed Consent Process and Documentation *ICF: Informed Consent Form, ICD: Informed Consent Document, LAR: Legally Authorized Representative; NDCT: New Drugs and Clinical Trials

No.	Domain	Key Components
1	ICF/ICD Content Quality	Purpose of the study clearly stated, voluntariness mentioned, risks explained in simple language, benefits described, alternatives mentioned (if applicable), confidentiality clause present, compensation for injury clause (as per NDCT), withdrawal rights explained, contact details for questions provided, language of the ICD appropriate (local/translatable)
2	Counseling Process	Verbal explanation provided to the participant, use of language understandable to the participant, time allotted for consent adequate (>10 mins recommended), opportunity given to ask questions, no coercion or undue influence documented, process done in a private setting (or appropriately documented)
3	Comprehension Assessment	Any method used to assess participant understanding (e.g., explain-back, quiz), participant asked to repeat key points in own words (if applicable), consent obtained after confirming comprehension
4	Documentation Standards	Participant’s/LAR’s signature/thumb impression present, date of signing clearly mentioned, investigator’s signature and date present, witness signature present (mandatory in NDCT for vulnerable participants), version number and date of ICF mentioned, IEC approval stamp/documentation attached with consent
5	Process Consistency with Guidelines	ICF consistent with ICMR 2017 format/template - ICF includes NDCT 2019-mandated statements (e.g., compensation, AV consent if applicable), training of the student in research ethics/GCP documented, audio-video consent performed (for vulnerable populations or clinical trials), consent re-taken if protocol amendments occurred

Each checklist item within these domains was scored as 1 if it was present and adequate (or not applicable to the study) and as 0 if it was absent or inadequate, giving a maximum possible score of 30. Based on the percentage of the total score obtained, performance was categorized as: Excellent (80-100%), Good (60-79%), Satisfactory (40-59%), and Poor (<40%).

Data was collected independently by two trained investigators and subsequently verified by a third reviewer. The identity of the postgraduate and the concerned broad specialty were concealed to protect privacy. Information about Domain 1 was extracted from the informed consent forms and documents (ICF/ICD) submitted to the Institutional Ethics Committee (IEC). In contrast, data for the remaining domains were obtained through direct observation of the consent process at the study site. Each eligible student was evaluated during a single site visit during the first patient recruitment. Initially, data were collected from the untrained cohort. Following analysis of their outcomes, a training intervention was conducted for the subsequent batch, which was then assessed using the same five domains to determine the impact of training (Appendices: Tables 5-7).

Training Intervention

The trained batch (2024-2025) underwent structured training as part of their foundation module during the first month of PG orientation. The training was conducted over three sessions totaling 9 hours and included interactive discussions and hands-on activities. The detail of the training modules is presented in Table 2. Evaluation was done at the first patient recruitment.

**Table 2:** Components of the Training Program ICD: Informed Consent Document; NDCT: New Drugs and Clinical Trials

Module	Content Covered
Regulatory framework	Lectures on ICMR 2017 guidelines, GCP, NDCT 2019 rules
Consent documentation	Preparation of ICFs and ICDs
Communication skills	Counseling techniques for obtaining valid consent
Participant comprehension	Methods for assessing understanding
Interactive learning	Role-play sessions and case scenarios
Procedural aspects	Demonstration of documentation and IEC submission

Statistical analysis

Data was entered into Microsoft Excel and analyzed using SPSS version 20 software (IBM Corp., Armonk, NY). Descriptive statistics (mean, standard deviation, percentages) were used to summarize the data. Comparative analysis between trained and untrained groups was conducted using the Chi-square test for categorical variables and the independent t-test for continuous variables. A p-value of <0.05 was considered statistically significant.

Ethical approval

Approval for the study was obtained from the scientific review committee (comprising of institute administrators) and the Institutional Ethics Committee (ESICMCH/SNR/IEC-F548/11-2023) of ESIC Medical College and Hospital, Hyderabad. The study adhered to the ethical principles outlined in the Declaration of Helsinki, ICMR National Ethical Guidelines 2017, NDCT Rules 2019, and Indian GCP Guidelines. Written IC was obtained from all the postgraduate students whose theses were included in the study. All data was anonymized, and all source documents were kept confidential as per IEC standard operating procedures.

## Results

A total of 60 postgraduate students were included in the study, with 30 students from the untrained batch (Intervention 5 and observation 25) and 30 students from the trained batch (Intervention 6 and observation 24). The ICFs, process, and documentation practice with each student’s thesis were reviewed and scored using a structured checklist comprising 30 items across 5 key domains.

Overall compliance scores

The mean overall checklist scores were significantly (p < 0.001) higher in the trained batch compared to the untrained batches. The detailed results are presented in Table 3.

**Table 3:** Overall Checklist Score among Untrained versus Trained Groups

Group	Mean Total Score (Out of 30)	Mean % Score	Grade Classification
Untrained (n = 30)	15 ± 3	51%	Satisfactory
Trained (n = 30)	26 ± 2	86%	Excellent

Domain-wise comparison

Across all five assessed domains, the mean checklist scores were consistently and significantly higher in the trained batch compared to the untrained batch (p < 0.001). In addition, the proportion of participants achieving an “excellent” rating was markedly greater among the trained group. Table 4 provides the detailed domain-wise results.

**Table 4:** Domain-Wise Checklist Score among Untrained (n=30) versus Trained (n=30) Groups

	Group	Mean Score ± SD	Participants Scoring ≥80%
Domain-1 (Score=10)	Untrained	6 ± 1	8%
	Trained	9 ± 1	95%
Domain-2 (Score=6)	Untrained	3 ± 1	7%
	Trained	5 ± 1	88%
Domain-3 (Score=3)	Untrained	1 ± 1	13%
	Trained	2 ± 1	92%
Domain-4 (Score=6)	Untrained	4 ± 1	12%
	Trained	5 ± 1	89%
Domain-5 (Score=5)	Untrained	3 ± 1	11%
	Trained	4 ± 1	86%

ICF/ICD Content Quality (score: 10): The trained group performed significantly better compared to the untrained batch (p < 0.001), with notable deficiencies in the untrained group, including inadequate mention of compensation clauses (38%), withdrawal rights (42%), and poorly articulated risk/benefit sections (30%).

Consent Process Quality (score: 6): Scores were significantly higher in the trained group compared to the untrained batch (p < 0.001). Common gaps in the untrained group were inadequate time spent on consent (42%) and limited opportunity for participant questions (36%).

Comprehension Assessment (score: 3): Trained participants scored significantly higher compared to the untrained batch (p < 0.001), while only 5% of the untrained group documented a comprehension check.

Documentation Standards (score: 6): Trained participants showed significantly better adherence compared to the untrained batch (p < 0.001), whereas deficiencies in the untrained group included missing the investigator’s signature/date (42%) and omission of the version number/date of the ICF (56%).

Regulatory/Ethical Compliance (score: 5): The trained group demonstrated significantly superior compliance compared to the untrained batch (p < 0.001). In contrast, the untrained group showed issues such as use of outdated consent templates (34%), absence of Audio-visual consent for vulnerable populations, and failure to retake consent following protocol amendments (0%).

Grading of overall performance

**Figure 1:**
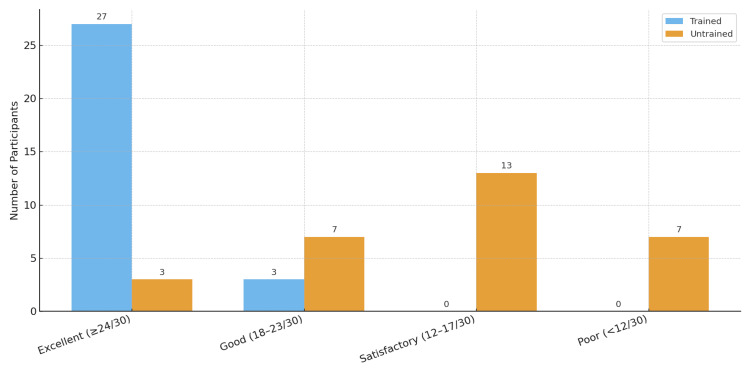
Grading of Overall Performance Among the Batches

In the untrained batch, the majority were classified as “Satisfactory” (13 participants, 43.3%). In contrast, in the trained batch, the majority of participants (27, 90%) attained an “Excellent” score. Grading of overall performance among the batches is presented in Figure 1.

## Discussion

This comparative study demonstrates a profound and statistically significant improvement in the quality of IC documentation and practices among postgraduate medical students following a structured training program. The findings strongly support the central hypothesis that formal education in research ethics is not only beneficial but essential for bridging the gap between theoretical guidelines and practical implementation. The untrained batch’s overall performance, graded largely as Satisfactory, aligns with existing literature highlighting consistent deficiencies in IC processes [2, 6, 11]. In contrast, the trained batch achieved an Excellent overall grade, underscoring the transformative potential of targeted educational interventions.

The domain-wise analysis offers granular insights into the specific areas where training had the most impact. The most dramatic improvements were observed in Comprehension Assessment and ICF/ICD Content Quality. The leap from 13% to 92% of students scoring ≥80% in assessing participant understanding is particularly noteworthy. This suggests that without specific training, the concept of verifying comprehension is almost universally overlooked, reducing consent to a mere signature. The training module dedicated to participant comprehension and the use of role-plays likely equipped students with practical tools like the teach-back method, moving beyond a checkbox mentality [14].

Similarly, the near-universal adoption of key content elements like compensation clauses and withdrawal rights in the trained group (95% scoring ≥80% in content quality) directly results from the regulatory framework and consent documentation modules. The training successfully addressed common deficiencies identified in other Indian studies [5,11], such as poorly articulated risks and missing mandatory NDCT statements. Furthermore, the significant improvement in Regulatory/Ethical Compliance, especially concerning audio-video consent and re-consenting after amendments practices that were virtually absent (0%) in the untrained group, indicates that the training instilled an understanding of dynamic and context-specific ethical obligations beyond a static form.

This study possesses several notable strengths that reinforce the reliability of its findings. Firstly, the use of a comprehensive checklist grounded in national (ICMR, NDCT) and international (ICH-GCP) guidelines ensures that the assessment was relevant and rigorous. Secondly, the strong methodology, incorporating simple random sampling, dual independent data collection with third-party verification, and a mix of document review and direct observation, minimizes bias and provides a holistic view of the IC process. The direct observation component is a particular strength, as it captures the practice of consent, which is often lost in audits based solely on paperwork [2, 7]. Finally, the clear, specific description of the 9-hour training intervention (Table 2) makes the study reproducible and provides a valuable template for other institutions.

Despite its strengths, several limitations must be acknowledged. The study was conducted at a single institution, which may limit the generalizability of the findings to medical colleges with different resources, mentorship cultures, or existing ethics curriculum. The non-blinded assessment of the outcomes, while mitigated by the structured checklist, presents a potential source of observer bias. There is also a possibility of cohort contamination, where informal knowledge transfer between the 2023-2024 and 2024-2025 batches could have occurred. Additionally, the study assessed immediate outcomes post-training, the long-term retention of these improved practices, and their translation into the students’ future careers as independent researchers, which remains unmeasured.

## Conclusions

The results of this study carry significant implications for the real-world landscape of clinical research ethics in India. They provide compelling evidence that a relatively brief, structured training program can dramatically elevate the ethical standards of research conducted by postgraduates. This is not simply an academic exercise; it directly impacts patient safety, autonomy, and trust in the medical research ecosystem. Based on these findings, we recommend integrating structured, hands-on training in research ethics and IC into postgraduate curricula, validating the model through multi-institutional studies, and conducting longitudinal follow-up to assess durability. Additionally, extending similar training programs to faculty and supervisors will help reinforce and sustain ethical research practices.

## Appendices

Template and checklist-based evaluation tool for informed consent process in PG thesis

Purpose

To objectively assess the quality and compliance of the informed consent process as practiced/documented by PG students in their thesis research.

Sections

1.     Informed Consent Form (ICF)/Informed Consent Document (ICD) Quality

2.     Process of Obtaining Consent

3.     Comprehension Assessment

4.     Documentation Standards

5.     Regulatory and Ethical Compliance

Scoring Format

Each item is scored as:

·        1 = Present and Adequate

·        1=  Not Applicable

·        0 = Absent or Inadequate

Grading:

·        Excellent: 80-100%

·        Good: 60-79%

·        Satisfactory: 40-59%

·        Poor: <40%

**Table 5:** Scoring Checklist NDCT: New Drugs and Clinical Trials; ICD: Informed Consent Document; ICMR: Indian Council of Medical Research; ICF: Informed Consent Form

Item	Criteria	Score (0/1)
Section 1: Informed Consent Form (ICF)/ICD Content (10 items)		
1.1	Purpose of the study clearly stated	/1
1.2	Voluntariness mentioned	/1
1.3	Risks explained in simple language	/1
1.4	Benefits described	/1
1.5	Alternatives mentioned (if applicable)	/1
1.6	Confidentiality clause present	/1
1.7	Compensation for injury clause (as per NDCT)	/1
1.8	Withdrawal rights explained	/1
1.9	Contact details for questions provided	/1
1.10	Language of the ICD appropriate (local/translatable)	/1
Subtotal Section 1	Out of 10	/10
Section 2: Process of Obtaining Consent (6 items)		
2.1	Verbal explanation provided to the participant	/1
2.2	Use of language understandable to the participant	/1
2.3	Time allotted for consent adequate (>10 mins recommended)	/1
2.4	Opportunity given to ask questions	/1
2.5	No coercion or undue influence documented	/1
2.6	Process done in a private setting (or appropriately documented)	/1
Subtotal Section 2	Out of 6	/6
Section 3: Comprehension Assessment (3 items)		
3.1	Any method used to assess participant understanding (e.g., explain-back, quiz)	/1
3.2	Participant asked to repeat key points in own words (if applicable)	/1
3.3	Consent obtained after confirming comprehension	/1
Subtotal Section 3	Out of 3	/3
Section 4: Documentation Standards (6 items)		
4.1	Participant’s/LAR’s signature/thumb impression present	/1
4.2	Date of signing clearly mentioned	/1
4.3	Investigator’s signature and date present	/1
4.4	Witness signature present (mandatory in NDCT for vulnerable participants)	/1
4.5	Version number and date of ICF mentioned	/1
4.6	IEC approval stamp/documentation attached with consent	/1
Subtotal Section 4	Out of 6	/6
Section 5: Regulatory and Ethical Compliance (5 items)		
5.1	ICF consistent with ICMR 2017 format/template	/1
5.2	ICF includes NDCT 2019-mandated statements (e.g., compensation, AV consent if applicable)	/1
5.3	Training of the student in research ethics/GCP documented	/1
5.4	Audio-Video consent performed (for vulnerable populations or clinical trials)	/1
5.5	Consent re-taken if protocol amendments occurred	/1
Subtotal Section 5	Out of 5	/5

**Table 6:** Final Scoring Summary

Section	Max Score	Your Score
Section 1: ICF Content	10	
Section 2: Consent Process	6	
Section 3: Comprehension	3	
Section 4: Documentation	6	
Section 5: Regulatory	5	
Total Score	30	/30

**Table 7:** Interpretation

Score (%)	Grade	Interpretation
24–30 (80–100%)	Excellent	Fully compliant with ethical standards
18–23 (60–79%)	Good	Minor improvements needed
12–17 (40–59%)	Satisfactory	Needs significant improvement
<12 (<40%)	Poor	High risk of non-compliance

·       

## References

[1] Shepherd V (2020). Advances and challenges in conducting ethical trials involving populations lacking capacity to consent: A decade in review. Contemp Clin Trials.

[2] Godskesen T, Björk J, Juth N (2023). Challenges regarding informed consent in recruitment to clinical research: A qualitative study of clinical research nurses' experiences. Trials.

[3] Pietrzykowski T, Smilowska K (2021). The reality of informed consent: Empirical studies on patient comprehension-systematic review. Trials.

[4] Kato R, Joseph R, Haule L, Kafuye M (2025). Readability of health research informed consent forms: Case of the National Health Research Ethics Committee in Tanzania. BMC Med Ethics.

[5] Chindhalore CA, Dakhale GN, Gajbhiye SV, Gupta AV, Khapeka SV (2024). Analysis of informed consent documents for compliance with ICMR guidelines for biomedical and health research. Perspect Clin Res.

[6] Jawa NA, Boyd JG, Maslove DM, Scott SH, Silver SA (2023). Informed consent practices in clinical research: Present and future. Postgrad Med J.

[7] Tam NT, Huy NT, Thoa le TB, Long NP, Trang NT, Hirayama K, Karbwang J (2015). Participants' understanding of informed consent in clinical trials over three decades: Systematic review and meta-analysis. Bull World Health Organ.

[8] Chatterjee K, Das NK (2021). Informed consent in biomedical research: Scopes and challenges. Indian Dermatol Online J.

[9] Paramasivan S, Davies P, Richards A (2021). What empirical research has been undertaken on the ethics of clinical research in India? A systematic scoping review and narrative synthesis. BMJ Glob Health.

[10] Gogtay NJ, Ravi R, Thatte UM (2017). Regulatory requirements for clinical trials in India: What academicians need to know. Indian J Anaesth.

[11] Khot A, Chindhalore CA, Naikwadi A (2024). Knowledge, attitude, and practices about research integrity and scientific misconduct among the faculty and medical postgraduates working in medical colleges in North Karnataka and central India: A cross-sectional online survey. Cureus.

[12] Nusbaum L, Douglas B, Damus K, Paasche-Orlow M, Estrella-Luna N (2017). Communicating risks and benefits in informed consent for research: A qualitative study. Glob Qual Nurs Res.

[13] Longstaff H, Lucas B, Schichter B, Murthy S, Flamenbaum J (2025). Core elements of consent documentation for clinical research in Canada: guidance for policy. CMAJ.

[14] Kamath YV, Shetty YC, Lanjewar IC, Kulkarni A (2025). Readability of informed consent documents and its impact on consent refusal rate. Perspect Clin Res.

[15] Shi Q, Luzuriaga K, Allison JJ (2025). Transforming informed consent generation using large language models: Mixed methods study. JMIR Med Inform.

[16] Kang S, Zhang J, Pang D, Yang H, Liu X, Guo R, Lu Y (2025). Impact of informed consent quality on illness uncertainty among patients with cancer in clinical trials: A cross-sectional study. Asia Pac J Oncol Nurs.

[17] Ong SW, Lee TC, Fowler RA (2024). Evaluating the impact of a SIMPlified LaYered consent process on recruitment of potential participants to the Staphylococcus aureus Network Adaptive Platform trial: Study protocol for a multicentre pragmatic nested randomised clinical trial (SIMPLY-SNAP trial). BMJ Open.

[18] Markman KM, Weicker NP, Klein AK, Sege R (2023). Community-engaged training in informed consent. J Clin Transl Sci.

